# Challenges and Drawbacks of the EU Medical System Generated by the COVID-19 Pandemic in the Field of Health Systems’ Digitalization

**DOI:** 10.3390/ijerph19094950

**Published:** 2022-04-19

**Authors:** Alexandra-Mădălina Țăran, Lavinia Mustea, Sorana Vătavu, Oana-Ramona Lobonț, Magda-Mihaela Luca

**Affiliations:** 1 Doctoral School of Economics and Business Administration, West University of Timisoara, 300223 Timisoara, Romania; alexandra.taran@e-uvt.ro; 2 Department of Finance, Faculty of Economics and Business Administration, West University of Timisoara, 300223 Timisoara, Romania; sorana.vatavu@e-uvt.ro (S.V.); oana.lobont@e-uvt.ro (O.-R.L.); 3 Department of Dentistry, Victor Babeş University of Medicine and Pharmacy, 300041 Timisoara, Romania; luca.magda@umft.ro

**Keywords:** European Union, method of vector quantization, digitalization, health, COVID-19, data mapping

## Abstract

The COVID-19 pandemic and the digitalization of medical services present significant challenges for the medical sector of the European Union, with profound implications for health systems and the provision of high-performance public health services. The sustainability and resilience of health systems are based on the introduction of information and communication technology in health processes and services, eliminating the vulnerability that can have significant consequences for health, social cohesion, and economic progress. This research aims to assess the impact of digitalization on several dimensions of health, introducing specific implications of the COVID-19 pandemic. The research methodology consists of three procedures: cluster analysis performed through vector quantization, agglomerative clustering, and an analytical approach consisting of data mapping. The main results highlight the importance of effective national responses and provide recommendations, various priorities, and objectives to strengthen health systems at the European level. Finally, the results reveal the need to reduce the gaps between the EU member states and a new approach to policy, governance, investment, health spending, and the performing provision of digital services.

## 1. Introduction

The COVID-19 pandemic has highlighted the pressing need for the healthcare systems of European countries to remain under the same values such as equality, accessibility, universality, quality of medical services, and equity [1].

Currently, the health systems of European countries are undergoing critical factors in the provision of safe and efficient healthcare, thus creating significant differences at the EU level on this issue. Considering the objectives of health systems, such as equity, safety, quality, and accessibility, the impact of digitalization on medical services and technological change may be an element associated with significant consequences for the delivery of healthcare and the future of health systems in the context of the COVID-19 pandemic. Most European Union member states (EU MS) receive high-level public support for government responsibility for health [2].

In recent years, European states’ various common strategies and plans have led to the determination of bold and transformative steps urgently needed to change health systems towards a resilient, sustainable, and efficient path. These credentials are even more important currently, in times of a global health crisis caused by the COVID-19 pandemic, which has brought critical risks and severe profound implications for health systems. Hence, more importance is given to how the population of a European Union perceives its health. In this regard, this growing concern for the health and safety of each person has received significant emphasis due to the development of the 2030 Agenda for Sustainable Development [3]. Therefore, various specific indicators have been developed to measure health, with a particular scope for creating and launching future sustainable public health policies.

Our study entails significant contributions and heightens the existing literature by offering a new comprehensive perspective on the challenges of the EU MS health systems generated by the specific implications of COVID-19 and the continuing process of digitization through the advanced method of vector quantization, the hierarchical clustering technique, and graphical representation.

The general objective of the paper was to assess the impact of technology integration on health systems and to measure public health under the effects of different COVID-19 indicators (cases, death, population receiving the first dose of vaccine, share of positive tests), emphasizing several groups of countries. Moreover, the inequalities of the EU member states in terms of each dimension are determined by a hierarchy of countries.

Public health programs and initiatives are based on an understanding of health behaviors and the context in which they occur. Therefore, interventions to improve health behavior can be best designed with an understanding of relevant theories of behavior change [4]. Our paper was developed based on the “social and behavioural science theory”—the transtheoretical model/stage of change model and the social ecological model—considering that long-term changes in health behavior involve multiple actions and adaptations over time within the decision-making process. Moreover, adopting innovation and phasing out obsolete practices are fundamental drivers of high-quality health care [5]. The two theories not only explain organizational and administrative behavior but also facilitate the understanding to develop more effective ways to influence and change behavior when the society is not ready to attempt changes and understand influential actors. Therefore, decision-makers should provide guidance for developing successful programs and large-scale health intervention social environments. Accordingly, the classic theory of the diffusion of innovation was also considered in our study because it identifies the primary influencers of the spread of new ideas, including innovation, communication channels, time, and social systems, especially during pandemic times.

After a concise and cursory introduction on the subject, the main finding from the literature is further described, centered on the importance of public health within the digitalization and COVID-19 pandemic framework at the level of the EU. Section 3 presents the data set used in the analysis and introduces the applied methodology. Section 4 and Section 5 enclose the main results and substantial discussions, followed by Section 6 with concluding remarks accompanied by guidelines, recommendations, and strategies for specific countries in the EU.

## 2. Literature Review

In the context of the COVID-19 pandemic, health systems show new inequalities across European countries, caused by dynamic change, adoption, and integration of technology and various devices needed in the field of digital health services, to which the digital skills of professionals and population are added, followed by lack of knowledge, credibility in the information transmitted, and digital literacy [6].

In recent years, various studies [7,8,9,10] have deepened the contributions to the specific dimensions such as health, digitalization, and COVID-19, employing different methodologies, with significant contributions to this scientific field (Tanahashi health system framework, analysis of policy trends on vaccine development and immunization programs, narrative review, and means of integrating technologies in healthcare). Studies have also been focused on the political organization of the countries and its impact in terms of the burden of COVID-19 infections at the beginning of 2020. Expecting countries with a more centralized political-administrative model (e.g., France, Portugal) to have a national health system better equipped in pandemic times, results evidenced that even decentralized countries (e.g., Germany, Italy, Spain, UK) transferred more power at the central level to reduce disparities across regions and ensure resource availability across the national territory [11]. Despite better coordination of healthcare resources for more centralized countries, evidence proved that this was insufficient to provide an effective testing strategy, mainly due to delayed processes. On the contrary, the testing strategy was better managed in decentralized states, as the regions could adapt their testing capacities quicker [12,13]. Economic growth also seemed to be an essential factor at the beginning of the pandemic because public administration focused on preserving limited medical resources and reducing unnecessary expenses in healthcare. In addition, the rapid spread of the COVID-19 amplified the pressure on hospitals and medical human resources in all countries, even in the most developed ones. Consequently, some countries or regions (especially those with high population density) faced a shortage of medical human resources and larger health inequalities, along with underfunding of the medical system and inefficient reactions from the political factors, which aggravated the COVID-19 spread [14,15]. Besides the common symptoms caused by the COVID-19 infection and its long-term effects, the pandemic augmented the mental health problems and reduced life satisfaction, even more for the younger population [16,17]. These problems were due to social isolation, economic fluctuations (related to reduced incomes, increased unemployment, limited activities, etc.), and reduced access to health services. These economic downturns increased the debt to GDP ratio at the national level, as governmental expenditures increased (due to healthcare and social assistance) and public revenues decreased due to lower taxes collected (reduced consumption and economic activity). However, these studies were based on national perspectives or a small group of countries.

The study conducted by Brătucu et al. [18] identified and analyzed the determinants of self-perceived health and the impact of the digitalization of the health system on the self-assessed health of European populations, based on a sample of 28 member states of the European Union. Employing a panel data regression method, the results indicated a positive correlation between how people assess their health, their ability to use the Internet to identify health information, and access to various applications to buy health-related items online.

Schmidt et al. [19] explored the digital transformation of the German health system, identifying critical factors in the adoption and implementation of this process, such as challenges, perspectives, and gaps, especially on how to improve digital literacy in digital health, as well as to increase the degree of participation and involvement of users.

Wendt et al. [2] analyzed the relationship between the institutional configuration and the pattern of public support for health systems based on the “Eurobarometer” survey of 14 European countries. The results revealed the existence of significant support from the state in the health system. Furthermore, the authors concluded that citizens’ perceptions of health systems are closely linked to the institutional commitment: (i) a low level of satisfaction with low-cost health care systems; (ii) a high level of satisfaction when health expenditure is considerable.

Malfatti et al. [20] state that, in the conditions of stopping the spread of the virus, the clinical activities were radically contained, being necessary to integrate the technology, such as innovative digital solutions in the medical system to provide various medical services.

The COVID-19 pandemic has revealed critical gaps in health systems while providing an opportunity to reflect on issues that may or may not have worked optimally, if action should be taken differently, and innovations that need to be integrated into the health care system. In this light, Negro–Calduch et al. [21] conducted a study through a questionnaire that assessed experiences regarding the performance of health information systems of different Member States to create specific corrective measures and specific directions regarding the expertise during the COVID-19 pandemic. The results evidenced the optimal functioning of the health information systems, which responded reasonably throughout the COVID-19 pandemic. However, the results outlined various critical issues of public health systems, such as lack of interoperability, insufficient resources, and outdated information technology.

Health systems are experiencing profound shocks due to the COVID-19 pandemic. Burke et al. [22] conducted a study based on the introduction ten years ago of a reform program named “Slaintecare”, aiming to provide prompt universal access to care. The authors examined whether or not the government’s response during the pandemic substantially contributed to the reform of the health system, the increase of the resilience of the health system, and the provision of universal medical assistance. The results proved that national policy documents and the intention to implement and develop the “Slaintecare” program were identified with a significant budgetary allocation. The results also provided an advanced understanding of the efforts made by other states’ governments to reform health systems during and after the COVID-19 pandemic and to develop public policies with the potential of transforming the health system.

Odone et al. [23] investigated the potential of applying digital tools to health systems, identifying the pillars for a successful common European strategy for digitizing health systems. The results highlight the benefits that the integration of technology in the medical field can bring benefits activated by various features of digital technology: (i) accuracy; (ii) automation process; (iii) forecast; (iv) data analysis; and (v) interaction.

Zimmermannova et al. [24] examined the benefits of the digitalization of medical devices during the COVID-19 pandemic through a thorough literature review, complemented with a regression and correlation analysis. The results indicated that the COVID-19 pandemic has significantly influenced resource distribution and hospital management, with digitalization being recommended as a tool for improving health systems.

To the best of our knowledge, none of the previous studies considered analyzing all three dimensions of health, digitalization, and COVID-19, at the same time and across all EU member states. Accordingly, our study assessed the impact of technology integration on health systems and evaluated public health under the effects of various COVID-19 indicators to emphasize the inequalities of the EU MS in terms of each dimension.

## 3. Materials and Methods

Typically, the main elements included in the objectives of European countries in terms of health systems are focused on several essential characteristics: efficiency, safety, quality, accessibility, and equity. Given the rapid evolution, both inside and outside the healthcare field, and the appearance and implications of the COVID-19 pandemic, the balance and optimization of the objectives have become an essential and continuous process for the quality and performance of health systems. 

Considering the above, three different methods have been applied to group the countries and to obtain a hierarchy of European countries according to certain specific indicators, as follows:

(i.) Analytical approach that consisted in applying data mapping that allows a hierarchy of sample countries regarding representative variables—graphical representation of the most representative indicators related to health, digitalization, and COVID-19 to capture the level of specific indicators in EU-27;
(ii.) To perform a vector quantization, K-means clustering is the selected method, by reporting to the latest available data;
(iii.) A hierarchical clustering technique for partitioning EU-27 countries into homogeneous clusters based on empirical similarity measures among those countries.

Thereby, to achieve the objective, the data set was configured based on the primary evidence found in the literature, including all the variables comprising our analysis for all the specific dimensions of health, digitalization, and COVID-19 indicators:

- Health representative indicators: “Life expectancy at birth by sex (years) (Life_EXP_birth)”; “Live births and crude birth rate (per 1.000 persons) (Live_births_crude_birth)”; “Median age (years) (MEDIAN_age)”; “Total fertility (live births per woman) (FERT_total)”; Health spending (Total, US dollars/capita) (HEALTH_spend)”;
- Digitalization indicators: “Human capital (weighted score (0 to 100)) (Human_cap_DESI)”; “Connectivity (weighted score (0 to 100)) (Connectivity_DESI)”; “Integration of digital technology (weighted score (0 to 100)) (Int_DigiTeh_DESI)”; “Digital public services (weighted score (0 to 100)) (DigiPubServ_DESI)”; “DESI Total (weighted score (0 to 100)) (Total_DESI)”;
- COVID-19 specific indicators: “Daily new confirmed COVID-19 cases per million people, 31 December 2020 (7-day rolling average) (Daily_new_cases_COV)”; “Daily new confirmed COVID-19 deaths per million people, 31 December 2020 (7-day rolling average) (Daily_new_deaths_COV)”; “Daily share of the population receiving a first COVID-19 vaccine dose (Daily_pop_FD_COV)”; “The share of daily COVID-19 tests that are positive, 31 December 2020 (Daily_positive_tests_COV)”.

More details on the indicators included in the data set are presented in the following Table 1:

The data were extracted for 2020 or the latest year available from Our World in Data for COVID-19 (daily, 31 December 2020) and some of the health variables (annual, 2020), Eurostat (annual, 2020) for other representative variables of health, and from Digital Agenda—European Commission for digitalization variables (annual, 2020). The descriptive statistics of the variables employed in our analysis are detailed in Table 2.

**Figure 1:**
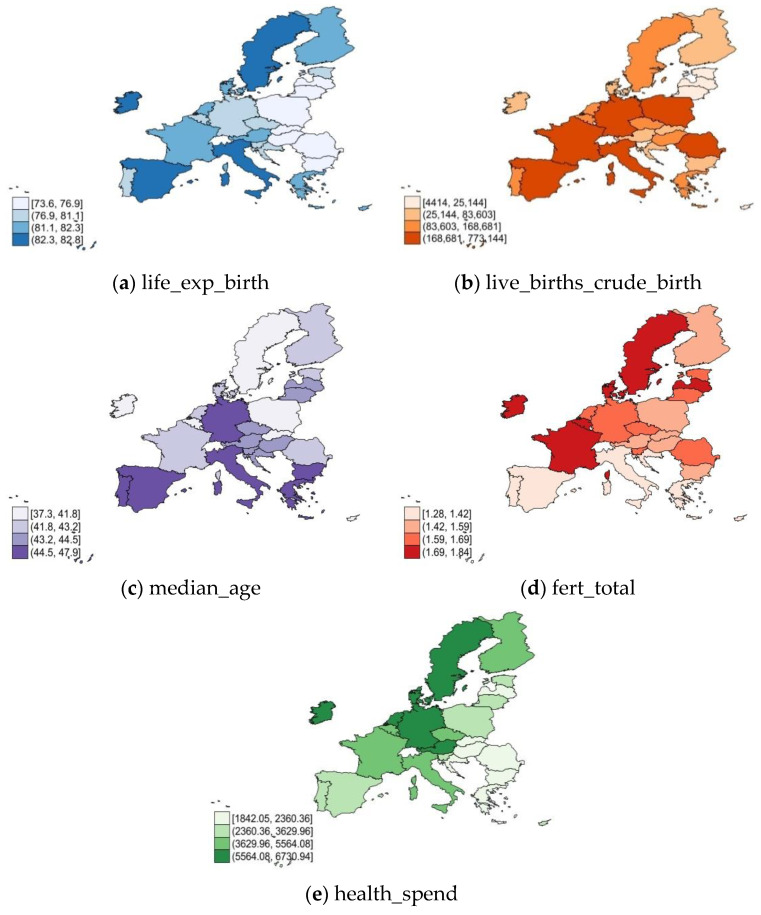
Health in EU-27, 2020: (**a**) life expectancy; (**b**) live births rate; (**c**) median age; (**d**) total fertility; (**e**) health spendings. Source: own process in Stata 17.

The main dimensions of health are graphically represented in Figure 1 across the EU in 2020. The results revealed the following: the higher life expectancy at birth (Figure 1a) occurs in Ireland, Italy, Spain, and Malta, but also in Sweden, associated with a high median age (Figure 1c) (except Ireland), and a strong fertility rate (Figure 1d). The most significant live births (Figure 1b) were demonstrated by the southern, western, and central states (Italy, Poland, France, Spain, Romania, Malta, Poland, and Germany). Germany, Austria, Netherlands, Luxembourg, and Belgium had the most substantial health spending. Along the same lines, the EU member states in CEE registered a low level of life expectancy at birth (Figure 1a), median age (Figure 1c), and total fertility rate (Figure 1d). On the other hand, from the EU, the lowest live births (Figure 1b) were in Lithuania, Latvia, and Estonia, and the lowest levels of health spending (Figure 1e) were identified in multiple countries (especially in Romania, Hungary, Slovakia, Croatia, Bulgaria, and Greece).

**Figure 2:**
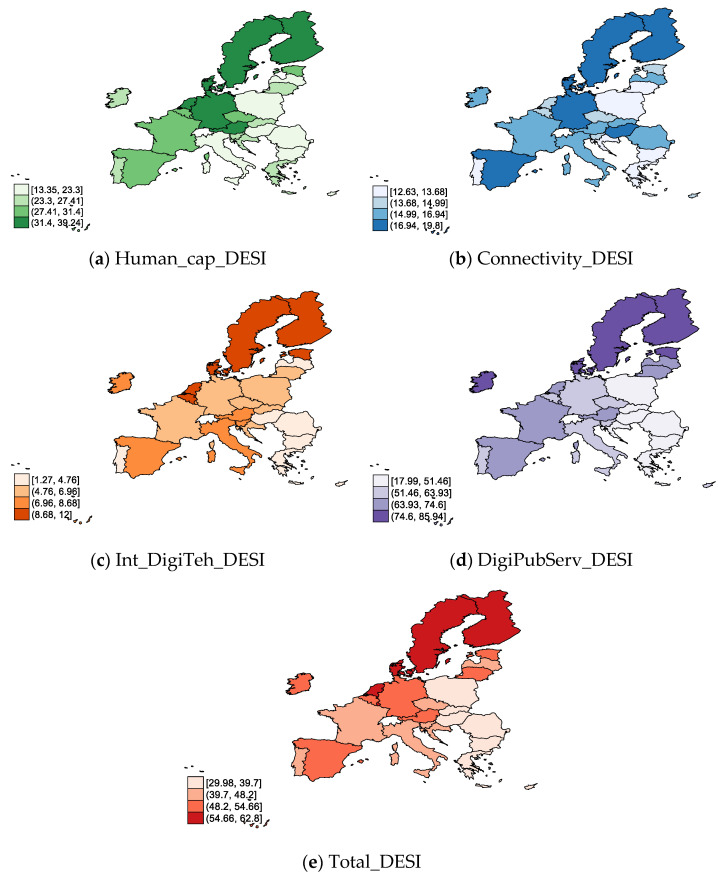
Digitalization in EU-27, 2020: (**a**) Human capital (by Internet user skills); (**b**) connectivity (by mobile broadband); (**c**) integration of digital technology (by digital intensity); (**d**) digital public services (by e-government) (**e**) DESI overall index. Source: own process in Stata 17.

In regard to the digitalization specific indicators (Figure 2), we noticed that in 2020 all of them were highly keen on EU MS, especially in the northern countries (Finland, Sweden, Denmark, Estonia, Ireland), and also in Estonia, Netherlands, Germany, and Austria. At the opposite end of the spectrum, a lower level of Human_cap_DESI (Figure 1a), Int_DigiTeh_DESI (Figure 1c), DigiPubServ_DESI (Figure 1d), and Total_DESI (Figure 1e) was observed in many countries (particularly in Romania, Hungary, Bulgaria, Poland, Lithuania, Greece, Croatia, and Slovakia). On the other hand, the lowest Connectivity_DESI (Figure 1b) tended to be highlighted in Poland, Bulgaria, Greece, Lithuania, Portugal, and Croatia.

**Figure 3:**
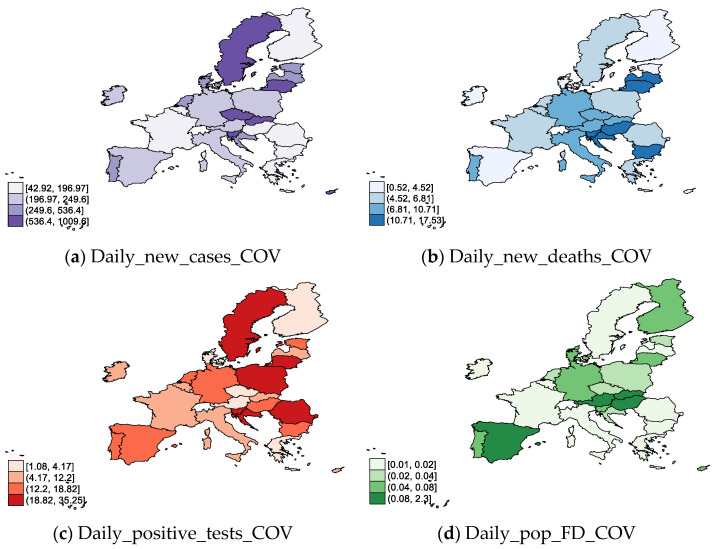
COVID-19 in EU-27, 2020 (31 December): (**a**) daily new cases; (**b**) daily new deaths; (**c**) daily positive tests; (**d**) daily share of the population receiving a first COVID-19 vaccine dose. Source: own process in Stata 17.

Considering the indicators related to COVID-19 (Figure 3) across the EU-27, on 31 December 2020, we can observe notable daily new cases of COVID-19 (Figure 3a) in Slovakia, Lithuania, Czech Republic, Slovenia, Sweden, Cyprus, and the Netherlands. Along with Slovenia, Croatia, Bulgaria, and Hungary, these countries also registered a high degree of daily new deaths (Figure 1b). Regarding daily positive tests of COVID-19 (Figure 1c), there are significant positive tests in many EU countries, except Ireland, Belgium, France, Cyprus, Malta, Finland, Germany, Greece, and Luxembourg. The highest Daily_pop_FD_COV on 31 December 2020 was observed in Poland, Slovenia, Lithuania, Croatia, the Czech Republic, and Romania. 

Data mining represents a technique by which it is possible to analyze large data sets, allowing the extraction and identification of previously unknown relationships and new structures. Information resulting from the data extraction process can be considered an added value for creating national or global strategies and at all stages of the decision-making process.

Cluster analysis is a significant area of research and the main topic of data mining [25]. Cluster analysis is a statistical method for data processing, a quantitative form of grouping data into categories based on their specific differences and similarities. It comprises many different algorithms and methods for grouping data and creating data groups [26]. Thus, objects belonging to one group are similar and different from objects belonging to others, each belonging to a specific group.

The K-means clustering algorithm will employ the KNIME application—the leading integration platform for reporting and data analysis needs. Open-source software for data analytics, reporting, and integration platforms can unite various components in machine learning and data mining with the concept of data diagrams.

**Figure 4:**
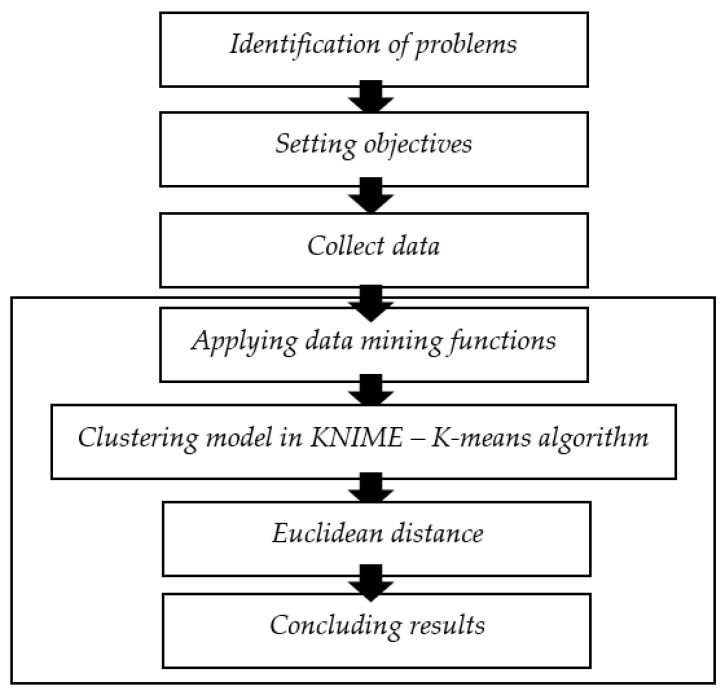
Research framework analysis techniques. Source: authors’ compilation.

The stages carried out started from problem identification, setting objectives, collecting data, processing data with the K-means clustering in the KNIME application, evaluating the quality of the clustering, and concluding the results as the flowchart in Figure 4.

**Figure 5:**
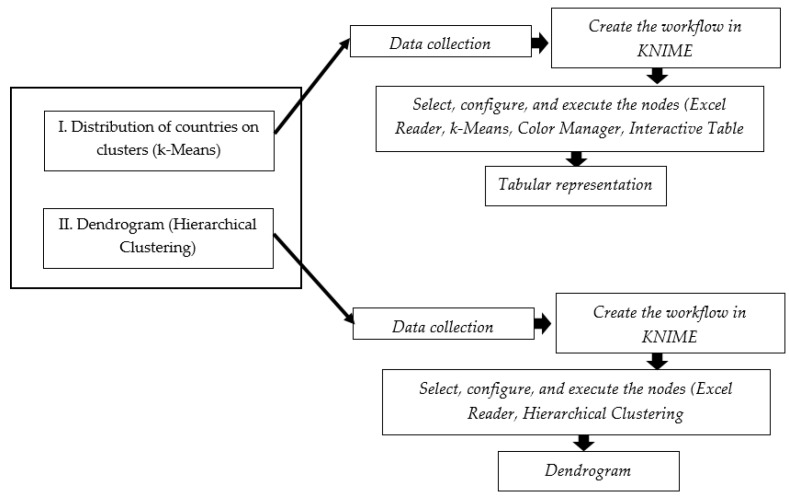
Research framework methods techniques. Source: authors’ compilation.

Moreover, the stages carried out in order to perform both types of hierarchies (dendrogram applied only in the second scenario, namely the ranking of the countries encompassing specific variables on digitalization—with hierarchical clustering and distribution of countries on clusters—and the K-means algorithm applied in all the three scenarios, such as: I. the scenario comprising health variables; II. the ranking of the countries encompassing specific variables on digitalization; and III. the scenario regarding certain representative variables on COVID-19) are presented in Figure 5.

For a fair comparison between EU MS and creating the clusters, the dataset was processed only for 2020. Then, the methodology used consisted of a vector quantization method, namely the K-means algorithm, through KNIME software. K-means clustering is one of the most popular methods employed in cluster analysis, in various fields. We opted for K-means clustering because it allowed us to run the algorithm in KNIME software, an intuitive platform with different machine learning components.

The stages of data clustering employing the K-Means algorithm are:

1. Determining the number of “k” clusters;
2. Determining the value of the midpoint (centroid) according to the number of clusters that have been selected.
  (1) $$C_{i}=min+\frac{\left(i-1\right)\times \left(max-min\right)}{n}+\frac{\left(max-min\right)}{2\times n}$$
  where:
  C_i_ = centroid of class “i”
  *min* = the smallest value of continuous class data;
  *max* = the largest value of discrete class data;
  *n* = number of discrete classes.
  1. Assigning each data to the center closest cluster:
    (2) $$D_{c}=\frac{\left(\sqrt{(Oi}-Ti\right)2}{{\left(Oj-Tj\right)}^{2}}$$
    where:
    D_c_ = distance of data to the cluster center;
    *O* = data records;
    *T* = data centroid.
  2. Determining the center of the new cluster (centroid):
    (3) $$C_{i}=\frac{O_{i}+\ldots +O_{n}}{\sum^{O}}$$
    where:
    C_i_ = centroid of class “i”;
    *O_i_* = value of data record to 1;
    *O_n_* = data record value to n;
    ∑^𝑂^ = number of data records.

Furthermore, the K-means clustering algorithm contains a complex process that involves measuring the distance between the data and each center of the groups. For this purpose, the Euclidean distance is applied and can be calculated by the following formula:

D(i,j) = (X1i− X1j) 2 + (X2i−X2j) 2 +…+ (Xki − Xkj)(4)

where:

D(i,j) = the distance between “i” and center cluster “j”;

Xki = the data to “i” on attribute data to “k”;

Xkj = the center point to “j” at attribute to “k”.

**Figure 6:**
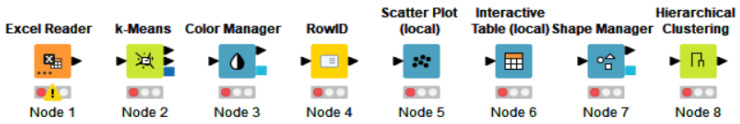
Representative elements (nodes) of a workflow in KNIME. (Node 1) spatial data; (Node 2) k-Means algorithm—perform clustering; (Node 3) assign colors to clusters; (Node 4) replace input data or create new row; (Node 5) create scatter plot; (Node 6) generate the table with afferent clusters; (Node 7) assign a shape to clusters; (Node 8) hierarchical—dendrogram. Source: authors’ compilation in KNIME analytics platform.

The advantage of employing KNIME software is represented by the opportunity to model each stage in machine learning with various programming languages. The nodes of a workflow clustering in KNIME include the EXCEL reader, rowID, k-Means, color manager, shape manager, scatter plot (local) and interactive table (local), hierarchical clustering, as shown in Figure 6. More details related to the workflow elements will be further presented.

The Node Excel Reader was the first node applied in the workflow, selected for the purpose of introducing data from a single file, so the node read and allowed the data introduced to be further processed. The node configuration included a series of features that allow the modification and preview of the data introduced. The execution part included the scanning of the uploaded file and determined the number of columns as well as their type, the final result being the table with data containing all the changes in terms of features and possible settings that were run along the process, until achieving the final form. In addition, the final table continued the settings realized and could be updated even after the initial settings were changed.

K-means represents the algorithm that establishes and sets the cluster centers for a predefined number of groups. Furthermore, it makes it possible to assign an exact data vector to a cluster, allowing a predetermined number of clusters, and employing Euclidean distance when running on the selected attributes to determine the distance between them. Before execution, the configured settings allowed, in addition to establishing the number of clusters, the selection of columns, i.e., of the variables on which we wanted to perform the grouping. Several attributes could be included or excluded from the analysis.

The Color Manager allowed for the assignment of different colors to both nominal and numeric columns. Moreover, it allowed for the selection of colors from a multitude of shades, so that we could differentiate the final clusters formed by the assigned colors. This color assignment feature was available within the group and also for each individual variable. In the case of a single observation selected, the color was assigned only to the minimum and maximum value, while in the case of selecting the group analysis, the colors were assigned according to the number of clusters.

The row ID enabled the creation of a new column, but also the replacement of the Row ID of the input data with the values of any other column, by means of the conversion of the values.

The Scatter Plot (local) allowed for the selection of observations from the database, creating a scatter plot for two selected attributes. The data points occupied a unique and specially designed space, depending on the values they held, being displayed with a different color, already set through the color manager, but also with a different shape, an option possible through the shape manager. The configuration allowed for the selection of variables, but also of certain characteristics related to the size of the points displayed in the scatter plot, or the background color or attributes of column X and column Y.

The Interactive Table (local) is the node through which the view of data through a table is possible. This node presented the final results containing the clusters preset by the K-means algorithm, each cluster having colors already established after the configured and executed filter from Color Manager. In fact, the Interactive Table also allows the attributes’ visualization, containing different columns: the columns existing from the beginning of the analysis, the paths, and the final output, i.e., the “S” column that continued the cluster name and number.

The Shape Manager represented the node through which the value of each attribute was assigned a different form. The major contribution of this node was represented by the fact that once the shapes of the analyzed attributes were assigned, the values could be distinguished by their shape when viewing the scatter plot, so the values in one cluster would have a different shape than the values of other clusters. Configuring this node continued the shape settings for every cluster.

Hierarchical Clustering allowed for the hierarchical grouping of input data. Hierarchical grouping was realized from the bottom-up or through agglomeration. The algorithm started with each data point as a single cluster, trying to combine the similar ones into several sub-clusters, until one reached a single cluster whose component includes all sub-clusters created. Essentially, the node had two ways of measuring the distance between observations: Euclidean distance and Manhattan distance. The configuration also allowed us to change the appearance of the dendrogram, with various options, such as the default and appearance settings. The entire hierarchy of the cluster was displayed via the dendrogram which, on the bottom side, continued all the data points of the analysis. The connection within the dendrogram was made only between the nearest data points, with the height of the connection suggesting the distance between them. The Y-axis, through the Y coordinates, showed us the hierarchy level, and through the X coordinates, the nominal values and the unique data points were projected.

The clustering implementation process was carried out in KNIME by employing the nodes arranged in the workflow. Furthermore, to create the network of elements that allow the creation and division of data into clusters, we applied a set of variables specific to each dimension analyzed. Thus, the model below is implemented to obtain a classification into clusters and a hierarchy of EU MS according to three different scenarios: (i) the scenario comprising health variables; (ii) the ranking of the countries encompassing specific variables on digitalization; (iii) the scenario regarding certain representative variables on COVID-19. 

For each scenario, the analyzed period covered the year 2020 or the latest available, taking into account both an analysis through the cluster modelling presented to the dendrogram that allowed for an intuitive hierarchy, but also through the K-means clustering algorithm that offered the possibility of partitioning the data set into “k” different groups, whose points did not overlap, and wherein each country belonged to a single group, thus having the possibility to observe the data points concretely in each group, directly generating the table of groups with the related countries.

## 4. Results

### 4.1. The Results for K-Means Clustering

A starting point in the cluster analysis was the creation of groups that contain EU countries in the context of health indicators. Thus, through a vector quantization method, it was possible to perform a cluster analysis that allows the association of countries according to specific values of the indicators, visualization, and intuitive grouping of observations.

#### 4.1.1. K-Means Clustering for Health

The method was possible to run through the KNIME platform, applying the cluster method and K-means algorithm, with a sample of five indicators representative of the health dimension [27,28].

Thus, the cluster implementation process was performed by KNIME software, considering nodes arranged in the workflow. The representative health indicators (Life_EXP_birth, Live_births_crude_birth, MEDIAN_age, FERT_total, and HEALTH_spend) were grouped in the excel format and introduced in the excel reader node, which was configured and executed with other KNIME elements, which allowed for the display of the results obtained after connecting the selected components in the workflow.

**Figure 7:**
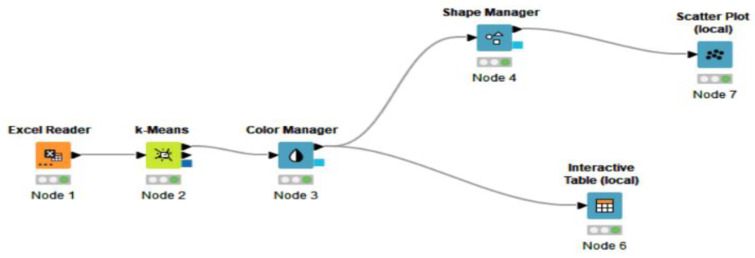
KNIME software elements (nodes) in the workflow regarding health indicators. Source: authors’ compilation in KNIME Analytics Platform.

Furthermore, Figure 7 highlights the elements chosen to create the clusters, and Figure 4 depicts the result of the process after connecting the components.

Workflow elements are essential in cluster analysis, being the characteristics on whose connection the final output depends. The connection of the elements contains the interactive table as the final stage that allowed us to visualize the clusters formed by the k-means algorithm and the distribution of the countries in clusters according to the chosen data points. In order to perform the analysis, it was necessary to create a database in an excel document, the data being recorded and adjusted through node 1. Furthermore, the K-means algorithm was selected to create the distribution of the clusters, allowing us to select a number of three clusters. The three clusters could be differentiated by introducing another node, the Color Manager, to determine the color of each cluster. Furthermore, all the nodes were connected to an Interactive Table in order to see the processed data in a tabular representation of the clusters.

**Figure 8:**
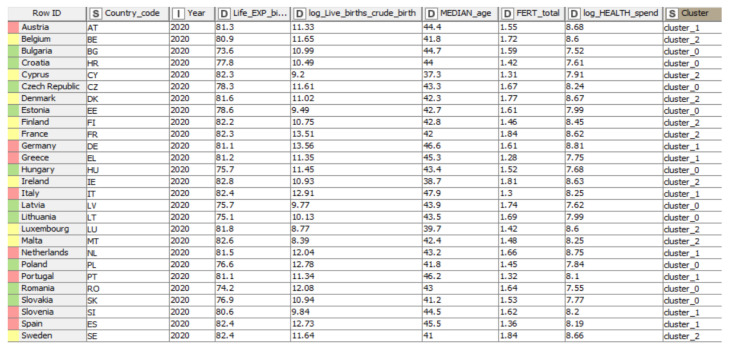
Countries displayed by clusters, based on health indicators. Source: authors’ compilation in KNIME analytics platform.

By employing the interactive K-means algorithm, the data set was partitioned into three distinct, predefined, and non-overlapping clusters. Each data point belonged to a single cluster. The results indicated different groups. The data points identified in a particular cluster were considered very similar; thus, each cluster was characterized by a different and unique data set. The results indicate that the sum of the square distance between the data points and the cluster centroid for the assigned data points is minimal. The Interactive Table allows the observation of a small number of variations within the clusters, the data points being more similar (homogeneous) within the same cluster. Hence, the tabular representation in Figure 8 allowed us to identify different columns of data, such as column “Row ID”, which contained information about the names of the analyzed states, column “I”, which indicated the year of the analysis, column “S”, which contained, in the first part, regarding column number two that indicated the country code afferent to the analyzed states, while column number nine, the one at the end of the table, showed the number of formed clusters, assigning to each country a specific cluster (0.1 or 2), and last but not least, the “D”-type column, containing both the names of the analyzed health indicators (the indicators being ordered as follows: “Life expectancy at birth by sex”, “Live births and crude birth rate”, “Median age”, “Total fertility”, and “Health spending”), and their related values.

Regarding the robustness of the groups, the variables presented approximate close values in terms of overall recorded values. The results were interpreted for the 2020 year for the EU-27 sample. Some variables were normalized by subjecting the data to the logarithmic procedure to compare the EU-27. 

Thus, life expectancy at birth by sex (years) had a maximum value of 82.8 years, a value related to Ireland, which belonged to group 2 (yellow). By contrast, the lowest value was recorded in Bulgaria, respectively, 73.6 years, the country part of group 0 (green). Furthermore, group 1 (pink) included values recorded between 80.6 and 82.4 years, at the average of the minimum and maximum values recorded by the indicator. Log live births and crude birth rate (per 1000 persons) recorded the full value of 13.56 (per 1000 persons) for Germany, which was part of group 1 (pink), and the minimum value was 8.39, attributed to Malta, a country included in group 2 (yellow). Median age (years) was identified as being the most favorable value in group 1 (pink), respectively, with 47.9 years for Italy. The opposite pole was Cyprus, with a value of 37.3 years, with group 2 (yellow) thus registering an unsatisfactory value. On the other hand, total fertility (live births per woman) contained values between 1.74 (Latvia) and 1.42 (Croatia) live births per woman for group 0 (green). Group 1 (pink) recorded values between 1.28 (Greece) and 1.66 (Netherlands) live births per woman, and group 2 (yellow) recorded values ranging from 1.31–1.84 live births per woman. Another variable identified was Log Health spending (US dollars/capita). Group 1 (pink) contained the maximum value of 8.81 total US dollars/capita (Germany) related to this criterion, on the contrary, finding Bulgaria with an unsatisfactory value of 7.52 (US dollars/capita).

#### 4.1.2. K-Means Clustering for Digitalization

As a starting point, we propose to determine the level of digitalization of the countries of the European Union for the 2020 year. In this sense, we compiled the data of dimensions of the DESI Index [29], indicators that allowed us to assess the level of DESI for UE-27, as follows:

1. Human capital;
2. Connectivity;
3. Integration of digital technology;
4. Digital public services.

Based on the study conducted by Stavytskyy et al. [30], where the authors applied a panel regression on the structural units of DESI, our study made a new contribution by regressing the specific dimensions of DESI as explanatory variables with the DESI overall index (DESI Total) as a dependent variable. Thus, to highlight the most significant contribution of the sizes in the DESI overall index and observe the particulate levels of each country in the EU-27, we proposed a regression analysis. In this sense, our model contained variables related to DESI; we found the apport of the following criteria: Human_cap_DESI, Connectivity_DESI, Int_DigiTeh_DESI, and DigiPubServ_DESI, designed based on the R square regression coefficients highlighted in Table 3.

According to Table 3, the coefficients indicate the most significant variables in determining the DESI overall index (DESI Total): Human_cap_DESI, Int_DigiTeh_DESI, and DigiPubServ_DESI, closely followed by Connectivity_DESI.

**Figure 9:**
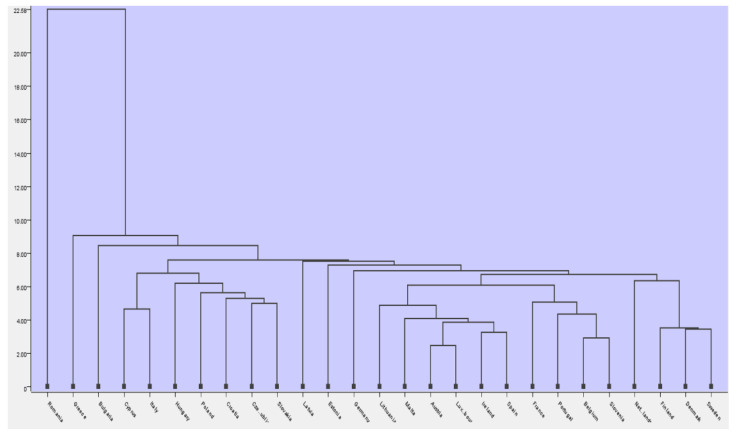
Dendrogram of clusters. Source: authors’ compilation in KNIME analytics platform.

Next, based on the variables that resulting from the most significant in the regression model, we created a hierarchical clustering for the 27 European states for 2020. The resulting clusters were ma following the application of hierarchical clustering in KNIME, using agglomerative clustering to group data into different clusters, using single linkage and Euclidian distance, resulting in the dendrogram presented in Figure 9.

The intuitive distance between the clusters is smaller as we approach the dendrogram’s minimum scale, between 4.00–22.58. Countries such as Romania, Greece, and Bulgaria presented the lowest values regarding the considered criteria (Human_cap_DESI, Int_DigiTeh_DESI, DigiPubServ_DESI, Connectivity_DES), being part of different clusters and having values that show significant differences compared to the digitalization average of the other countries. A significant distance between the groups indicates that their differences are large. Despite the efforts of the governments of Romania, Bulgaria, and Greece, they face major difficulties and challenges in terms of digital technology integration.

**Figure 10:**
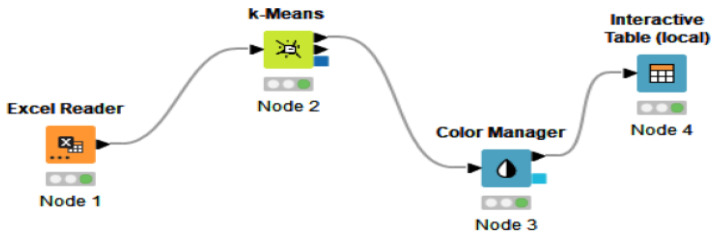
The workflow of KNIME with nodes regarding digitalization indicators. Source: authors’ compilation in KNIME analytics platform.

Subsequently, we applied the K-means algorithm to five selected indicators of the digitalization dimension. We applied the same model as in the section above, creating a workflow with the necessary elements for analysis (Figure 10), considering the digitization indicators, as follows: Human_cap_DESI, Connectivity_DESI, Int_DigiTeh_DESI, DigiPubServ_DESI, and Total_DESI.

**Figure 11:**
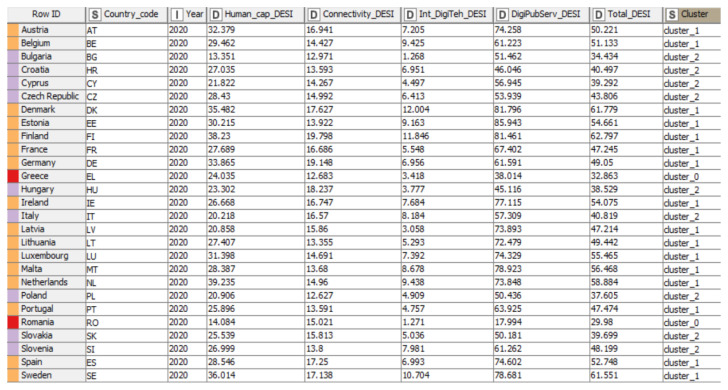
Distribution of countries based on digitalization indicators. Source: authors’ compilation in KNIME analytics platform.

The excel reader node allowed us to enter data in KNIME, and the K-means algorithm was applied via the node. Furthermore, the different clusters in the interactive table contained different colors, differentiating the groups, which could be created and viewed by employing a color manager. Moreover, Node 1 was introduced in order to read the data and Node 2 allowed us to select the number of clusters by configuring the node. After configuring and executing both nodes (Excel Reader and k-Means), in order to obtain a tabular representation of the country groups (clusters), we introduced the Color Manager node, which made it possible to set the colors for the groups, and then we connected this element to an Interactive Table in order to obtain Figure 11.

The clusters obtained from the application of the K-means algorithm were distinct, and the results revealed that the data points did not overlap, being unique and belonging to a single group. The number of variations between the 3 clusters was relatively insignificant, the observations (data) in each cluster being homogeneous. 

According to Figure 11, the tabular representation highlighted the analyzed data in several different columns. Thus, the column “Row ID” indicated the name of the states, while column “S” referred to both the code name of the countries (column number two) and the name and the cluster of which the analyzed countries were part (column number nine). Moreover, we could observe the year of analysis in column “I”, but also the columns marked with “D” that indicated the values of the analyzed digitalization indicators (“Human capital”, “Connectivity”, “Integration of digital technology”, “Digital public services”, and “DESI Total”).

Human_cap_DESI (the weighted score (0 to 100)) presented different values for each group, as follows: for group 0 (red) values between 24.03 (Greece) and 14.08 (Romania), secondly for group 1 (orange) recorded values for the range 20.85 (Latvia)—39.23 (Netherlands), and last but not least group 2 (purple) with the highest value of 28.43 (Czech Republic), and the lowest value being 13.35 (Bulgaria). 

Further, Cluster 1 (orange) registered the highest value of the criterion Connectivity_DESI (weighted score (0 to 100)) (Finland: 19.80), while the lowest value was recorded in cluster 2 (purple) (Poland 12.63). 

For all groups, the criterion Int_DigiTeh_DESI (weighted score (0 to 100) registered a maximum value of 12.00 (Denmark)—cluster 1 (orange), and a minimum value of 1.27 (Romania)—cluster 0 (red).

The highest values of the criterion DigiPubServ_DESI were observed in cluster 1 (orange) (Estonia: 85.94), and at the same time, the lowest value was registered in cluster 0 (red) (Romania: 17.99). 

As a general overview, the Total_DESI registered a maximum value of 62.79 in cluster 1 (orange)—Finland, and a minimum value of 29.98 in cluster 0 (red)—Romania. Therefore, the cluster analysis showed significant disparities within the three clusters between the EU MS, which was also found in the study conducted by Kinnunen et al. [31].

#### 4.1.3. K-Means Clustering for COVID-19

**Figure 12:**
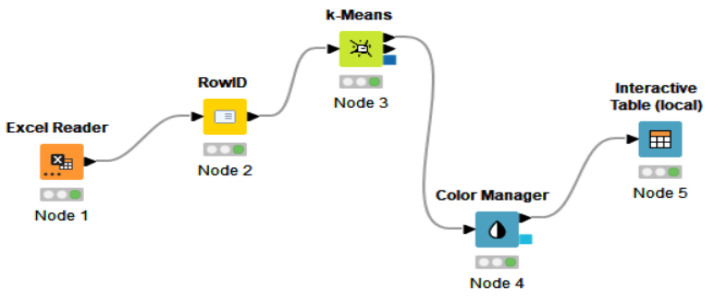
KNIME workflow with elements regarding COVID-19. Source: authors’ compilation in KNIME analytics platform.

The nodes for K-means clustering are entailed in Figure 12, selected, and connected in the KNIME workflow. The first step in performing K-means clustering in KNIME was to create the workflow by connecting the necessary elements. In addition, the first essential element of the analysis was Node 1 through which the data were entered into KNIME software, configured, and executed. Furthermore, the first node and the “RowID” node were connected to Node 3, which allowed us, with the help of the configuration settings, to establish the number of clusters. However, to establish the color of each cluster, we applied node 4, and then we connected it to the last node of the analysis, namely the Interactive Table. Moreover, the cluster analysis was performed for the COVID-19 dimension, considering the following indicators: Daily_new_cases_COV, Daily_new_deaths_COV, Daily_positive_tests_COV, and Daily_pop_FD_COV.

**Figure 13:**
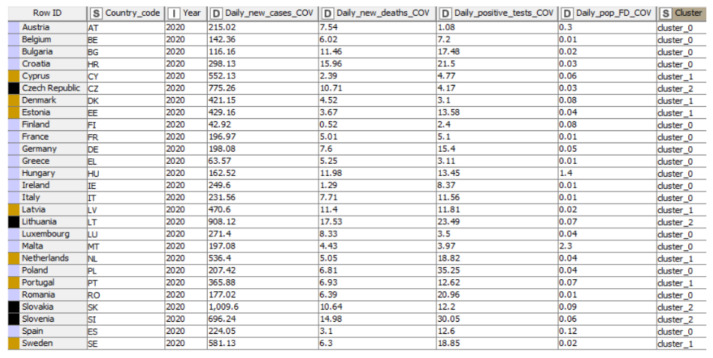
Distribution of countries into clusters, based on COVID-19. Source: authors’ compilation in KNIME analytics platform.

In Figure 13 each of the selected elements, called nodes, led to the tabular representation into clusters (column nine—“S”) of the analyzed countries (column “Row ID”) in the selected year (column “I”). Furthermore, the “D” type of columns indicated the name and the values of the COVID-19 representative indicators (“Daily new confirmed COVID-19 cases per million people”, “Daily new confirmed COVID-19 deaths per million people”, “Daily share of the population receiving a first COVID-19 vaccine dose”, and “The share of daily COVID-19 tests that are positive”).

Through K-means clustering, the data set was grouped into three clusters of different colors (blue, brown, and black). Data points were found in a single group with appropriate values. The formed groups were differentiated either by assigning a new column that specifies the name of the cluster or by inserting the color manager node among the selected elements. The results were presented through an interactive table, connected and linked to the rest of the workflow elements, resulting in the formation of three groups of countries. The results of the distribution of the countries in groups showed similar values within the created clusters, thus confirming the robustness. Regarding Daily_new_cases_COV, the values varied between 1009.6–42.92, the maximum value registered by Slovakia (1009.6—cluster 2), and the minimum of 42.92 (Finland—cluster 0). Furthermore, Daily_new_deaths_COV recorded the maximum value in cluster 0, a value assigned by 0.52 to Finland. In contrast, the maximum value was 17.53 (cluster 2—Lithuania). Daily_positive_tests_COV was characterized by the maximum value assigned to cluster 0 (Poland—35.25), and the minimum value was 1.08 (Austria—cluster 0). For the three clusters, the minimum value was 1.08, registered in Austria. Next, Poland (cluster 0) noted a maximum value of 35.25. Regarding Daily_pop_FD_COV, the highest degree of this criteria was significantly demonstrated by Malta (cluster 0) with the value of 2.3. At the opposite end of the criteria values, we observe Belgium, France, Greece, Hungary, Ireland, Italy, and Romania, the countries that registered the lowest value in cluster 0 (value 0.1).

## 5. Discussion

Our main findings stated that the vulnerability of health systems of the EU-27 member states has been highlighted by the COVID-19 pandemic differently in the analyzed groups, with profound implications in various areas, such as resilience, economic progress, health, social cohesion, sustainability, and trust in government.

The results of the clustering following the application of the K-means algorithm in the KNIME analytics platform regarding the specific indicators of the health dimension are pointed out in Table 4.

Thus, life expectancy at birth by sex (years) had a maximum value of 82.8 years, a value related to Ireland, which belonged to group 2 (yellow). In Ireland, this value was justified by a mix of improvements in many essential characteristics of people’s lives, such as: wealth and income, nutrition, educational attainment, behavior, public health, and medicine. By contrast, the lowest value was recorded in Bulgaria, respectively, at 73.6 years, the country being part of group 0 (green). Instead, we found Bulgaria, whose values may also be low due to the significant gaps in the provision of the population in terms of outpatient care and underdeveloped preventive actions. Furthermore, group 1 (pink) included values recorded between 80.6 and 82.4 years, being at the average of the minimum and maximum values recorded by these criteria. 

Live births and crude birth rate (per 1000 persons) record the maximum value of 13.56 (per 1000 persons) for Germany, which was part of group 1 (pink), and the minimum value was 8.39, attributed to Malta, a country included in group 2 (yellow). Germany gained the first position in cluster 1 due to many signs of progress in increasing the number of live births. Malta faced low values regarding this indicator due to the presence of certain health factors that can lead to a decline in the value of this criteria.

Median age (years) was identified as having the most favorable value in group 1 (pink), respectively, at 47.9 years for Italy. At the opposite pole was Cyprus with the value 37.3 years, with group 2 (yellow) thus registering the lowest value. The resulting values categorized countries, both Italy and Cyprus, in terms of exposure to the challenge of an aging society.

On the other hand, total fertility (live births per woman) contained values between 1.74 (Latvia) and 1.42 (Croatia) live births per woman for group 0 (green). Group 1 (pink) recorded values between 1.28 (Greece) and 1.66 (Netherlands) live births per woman, and group 2 (yellow) recorded values ranging between 1.31–1.84 live births per woman. The highest and lowest values may be due to factors in various fields, such as health, economic, cultural, political, and social. In addition, among the factors that most affect total fertility can be found the attitudes, female’s age, the high costs of raising a child, and employment problems. 

Another variable identified was represented by health spending (US dollars/capita). Group 1 (pink) contained the maximum value of 8.81 US dollars/capita (Germany) related to this criterion. Germany has the highest value for the criterion, being above the EU-27 average. Although the COVID-19 pandemic has identified gaps and highlighted health challenges, the German healthcare system is among the most performant in the EU due to its wide range of benefits and the provision of high-quality and efficient healthcare services. On the contrary, Bulgaria registered the lowest value of 7.52 (US dollars/capita), facing various problems in the health system, but probably an improvement of health reforms would boost health spending.

In the next section, we performed an analysis based on digitization indicators, with the countries being divided into different clusters, and a hierarchy of them could be observed in each indicator. The results obtained are presented in Table 5.

Human_cap_DESI (weighted score (0 to 100)) presented different values for each group, as follows: for group 0 (red) values between 14.08–24.03. In this case, the highest value was recorded by Greece due to the implementation of a strategy for digital transformation, which aimed to design and implement the objective of providing a high level of digitalization. Therefore, Greece also resumed coalitions to attract partners to build the plans needed to address internet user skills gaps. On the other side of the ranking of European Union MS in cluster 0 (red) was Romania with the lowest value, despite many projects that tend to stimulate and improve the level of internet user skills, with efforts needed in different directions of human capital. Secondly, for group 1 (orange), the recorded values ranged from 20.85 to 39.23. Latvia had the lowest value, with various low indicators in the group analysis, including internet user skills. Moreover, the level of internet user skills was below the average of the analyzed countries, caused by the problem of the non-existence of a specific strategy regarding the development and improvement of internet user skills. In the Netherlands, we observed the highest value. We also had a leading position in the ranking of countries in terms of Internet user skills, with a value far above the average of the countries analyzed at the level of clusters. The value recorded was due to the high importance of Internet user skills, a critical objective that underlines the digital strategy adopted and implemented in the Netherlands. Last but not least, group 2 (purple) had the highest value of 28.43, and the lowest value was 13.35. The resulting values indicated that the Czech Republic registered the highest values. The recording of such values may be due to the strategies focused on developing internet user skills, especially the critical pillars of education, respectively teachers, who can ensure the updating of school curricula. Moreover, Bulgaria registered the lowest values due to reforms that do not fully include concrete objectives of digital transformation. 

Further, Cluster 1 (orange) registered the highest value of the criterion Connectivity_DESI (weighted score (0 to 100)). Finland registered the highest value, with 19.80 being due to the excellent performance in terms of mobile broadband (inclusive ubiquitous 4G and 5G coverage with high household access). In contrast, the lowest value was reported in cluster 2 (purple) by Poland with 12.63. The value could be mainly attributed to the geographical conditions representing the main difficulties in implementing mobile broadband.

For all groups, the criterion Int_DigiTeh_DESI (weighted score (0 to 100)) registered a maximum value of 12.00 reported by Denmark, the leader of cluster 1 (orange), with a score of the digital intensity at a very high level, above the average of the analyzed countries. Furthermore, cluster 0 (red) recorded the minimum value of 1.27 by Romania due to the lack of specific measures to stimulate the spread of the benefits of digital implementation and standards regarding the digitalization of SMEs.

The highest values of the criterion DigiPubServ_DESI were observed in cluster 1 (orange) by Estonia with 85.94—ranks first place in cluster 1 on e-government, due to a well-developed e-government system providing services online. At the same time, we had the lowest value in cluster 0 (red) recorded by Romania with 17.99. The value registered was based on the problem that has been present for several years regarding the lack of interoperability of IT systems in public administration. Moreover, the problematic situation and the emergence of COVID-19 revealed the existing gaps in e-government and e-health in Romania. 

As a general overview, Total_DESI registered a maximum value of 62.79 in cluster 1 (orange)—Finland, and a minimum value of 29.98 in cluster 0 (red)—Romania. Therefore, the cluster analysis evidenced significant disparities within the three clusters between the European states, which was also found in the study conducted by Kinnunen et al. [31], which listed Romania as one of the countries with the lowest level regarding digitalization. In addition, the study highlighted the gap between the most and the least advanced digitalized countries.

The last part contained a cluster analysis for the COVID-19 criteria, where the countries were divided into different clusters according to values registered by indicators (Daily_new_cases_COV, Daily_new_deaths_COV, Daily_positive_tests_COV, and Daily_pop_FD_COV). The distribution is presented in Table 6.

The main results showed that COVID 19 virus had an infection rate and a high rate of spread at the end of 2020 (December 31), with many daily new confirmed cases (Daily_new_cases_COV) in Cyprus, Lithuania, Slovakia, Slovenia, Sweden, Czech Republic, and COVID-19 positive tests (Daily_pop_FD_COV) in Poland, Slovenia, Lithuania, Romania, Bulgaria, and Sweden. Furthermore, the government should take measures to strengthen the capacity and resilience of health systems to respond immediately and effectively to everyday challenges, as the number of COVID-19 positive tests (Daily_positive_tests_COV) at the end of the year 2020 (December 31), a challenge faced by countries such as Belgium, France, Ireland, Romania, and Greece. European Union member states are also launching vaccination campaigns against COVID-19. Still, daily new confirmed deaths (31 December 2020) in Lithuania, Slovenia, Slovakia, Bulgaria, and Hungary present a trend that continues to rise, especially among older people. Our results complement the research of Rizvi et al. [32], who made a cluster analysis by considering only two of the variables analyzed within this paper. The results also attested high values of COVID-19 new cases and deaths in the analyzed clusters.

## 6. Conclusions

The progress suggested by this research evidenced the significant discrepancies of European countries in terms of health, digitalization, and COVID-19, and the importance of increasing the degree of digitization in health systems, especially during the pandemic when health systems are under pressure. This endeavor complements previous contributions on this subject, providing new evidence and implications of the COVID-19 pandemic in need of accelerating the digitalization of health systems. The particular focus of this research was on digitalization, given the critical position of this dimension in terms of the objectives of the European Commission. The methodological approach applied the K-means clustering to group the countries into different groups and the values of the analyzed criteria had similar and appropriate values, depending on three different dimensions that were interconnected: health, digitalization, and COVID-19. On the other hand, hierarchical clustering was also applied according to the most significant criteria resulting from the regression analysis for the digitalization dimension. 

This overall approach was necessary for the process of observing the situation of each country in the European Union, thus successfully managing to obtain a hierarchy of countries, grouping them into clusters according to the values of the criteria analyzed, as well as the smallest and the higher value recorded for each criterion in each EU member state. The results indicate significant variations across EU countries along the three dimensions analyzed: health, digitalization, and COVID-19, and EU countries thus need much closer cooperation. Countries with health care systems less affected by the COVID-19 pandemic could coordinate states that are more concerned, advancing their ability to respond more efficiently to health systems’ barriers and challenges. Moreover, another consideration to be adopted by the governments of European states is strengthening public health policies.

The main findings at the level of EU MS revealed that countries such as Bulgaria, Estonia, Romania, Croatia, Estonia, Czech Republic, Hungary, Latvia, Poland, Lithuania, and Slovakia need significant improvements in all five criteria of health dimension. Furthermore, these countries also belong to the cluster with the lowest values in terms of health. In a more advanced position, countries such as Austria, Germany, Greece, Italy, Netherlands, Portugal, Slovenia, and Spain could receive help from countries with a high level of health, thus reaching an optimal level of functioning of health systems. Regarding Ireland, Belgium, Cyprus, Denmark, Finland, France, Luxembourg, Malta, and Slovakia, the ranking places them in the cluster with the highest health representative indicators.

Significant gaps were identified concerning the level of digitalisation of EU MS countries, too. Although European Commission programs aimed at policy initiatives that significantly impact digital transformation, the overall level of digitalization was shallow in most European countries. In this light, Romania and Greece had a level below the European Union average, being very weak in terms of the overall adoption and progress of the DESI index. Moreover, countries such as Bulgaria, Croatia, Cyprus, Hungary, Czech Republic, Poland, Slovakia, and Slovenia also face a relatively low integration of the four dimensions of DESI. Overall, the highest digitalization average in terms of DESI implementation and operation is recorded by the cluster that contains many countries, as follows: Finland, Denmark, Ireland, Netherlands, Germany, Portugal, Spain, Austria, Belgium, Estonia, France, Latvia, Lithuania, Malta, and Sweden. 

The digitalization of medical services must be a primary objective of all European states, and public health policy and health spending are needed to support this process in the long term. These results demonstrate that health and digitalization are two fundamental pillars and essential components that can strengthen the health systems of EU MS in the context of the COVID-19 pandemic.

On the other hand, the COVID-19 pandemic highlighted the gaps, problems, and vulnerability of health systems and was also an essential factor in advancing ICT in the medical field. The acceleration of the digitalization of health systems was mainly due to the COVID-19 pandemic. The lockdown period highlighted the need for a digitalized health care system, which made it impossible to provide medical treatment, consultation, and services for a long time right from the beginning of the pandemic.

Concerning COVID-19 representative indicators on 31 December 2020, regarding daily cases and deaths, we identified countries such as Slovakia, Lithuania, Czech Republic, Slovenia, Sweden, Cyprus, Hungary, Latvia, and Bulgaria as the most severely impacted by the pandemic. Spain, Slovakia, Finland, Denmark, Portugal, and Lithuania had the highest proportion of the population vaccinated with the first dose. A low proportion of people receiving the first dose was registered in Romania, Greece, Belgium, and France. The virus’s rate was high in Poland, Slovenia, Lithuania, Croatia, Czech Republic, and Romania given the rate of infection and spread in the number of positive tests. Therefore, a joint agreement had to be developed at the EU level on public health, as vaccination was expected. In fact, in stopping the spread of the virus, a joint effort was made by EU states to limit the virus, including vaccination certificates, test plans, wearing a mask, and other public health measures. 

Furthermore, the COVID-19 pandemic continues to have negative consequences not only on health systems but also on society [33]. In addition to the first-order effects, related to the virus (infections, deaths, post-partum effects of COVID), we also added mental health effects of lockdown, and several key social influences in addition to health sector, particularly on employment and education. The unemployment levels increased, especially in the hospitality sector, as the food and drink services industry has faced unprecedented change due to pandemic. The companies were forced to invest and accelerate the trend of digitalization, introducing online ordering and delivery services. According to the Office for National Statistics in the UK, approximately 45% of the people who were infected with COVID-19 experienced long-term symptoms (after more than a year from infection), and around two-thirds declared their day-to-day activities were greatly affected. In addition, there was a large psychological toll as the number of people with anxiety almost doubled during the first wave of the pandemic. The younger population was even more affected by the greater financial uncertainty, job loss or increased childcare responsibilities during online education. Other behavioral aspects affected during the first wave of the pandemic were related to the worsening diet and reduced physical exercise. Fortunately, over time, the pandemic shock made people choose healthier food and exercise more, but these increased the costs of living. Children’s habits in general, and even more for children from families with lower incomes, were more affected in terms of the food consumed, sedentariness or socializing with friends, as schools are a source of exercise and more nutritious food. 

Certainly, the pandemic sped up the digitalization process in terms of communication, online consultations, telemedicine, and ensuring a remote patient monitoring. However, there is still a gap in the automation technology in hospitals, as the system needs drastic improvements in communication among caretakers, with the continuous update and access to patient information. This leads to future research considering the level of investments realized in public hospitals across countries, based on the level of public revenues for funding the healthcare system. In addition, future research would require more impactful evidence based on a larger dataset, with several years considered. To generalize the potential advantages and the inequalities across countries, a larger quantitative sample would be appropriate to extend our analysis. Romania, Bulgaria, and Greece were evidenced as countries facing major difficulties and challenges in terms of digital technology integration, while Nordic countries (Finland, Denmark, and Sweden) are top countries in the digitalization of healthcare. However, with reference to the COVID-19 indicators, the first set of countries faced a much lower level of daily new cases compared to Denmark or Sweden. The daily share of the population receiving a first COVID-19 vaccine dose was very high in countries such as Finland and Denmark, compared to Romania or Greece, which also makes us think that the analysis over a couple of years (2020 and 2021) would probably emphasize even more differences across countries, through clusterization, in terms of healthcare digitalization and COVID-19 implications.

The main findings can sustain policymakers in the decision-making process to control the COVID-19 pandemic. A problem during the pandemic period was the lack of trust in government decisions and public sector governance in general. A recent study evidenced that under conditions of higher trust in government and in interpersonal relations (similar to the levels registered in Denmark) the global infection rate could have been reduced by 40% [34]. In addition, it was evidenced that social disparities and low interpersonal trust are related to income inequality, and therefore, countries with higher inequalities in terms of social strata and income distribution were more affected by the pandemic. Under the current conditions, improving health systems remains the key to enhancing, maintaining and ensuring public health and economic growth, the research supports a series of recommendations/guidelines/objectives that could be implemented and considered by the policymakers and public institutions within the European Union member states, namely: (i) the government must support many more Europeans to vaccinate rapidly to avoid many infections and to stop the emergence of new variants of COVID-19; (ii) further, a common strategy of EU concerning the need to help the rest of the world to get vaccinated too, by exporting and donating vaccines in low-income and middle-income countries; (iii) amplified typical EU MS response to the pandemic outbreak, by taking resolute action to reinforce the public health sector and to mitigate the social, economic impact; and (iv) national response of the EU MS to provide objective information about the spread of COVID-19 and direct efforts to contain the virus.

As mentioned before, the lack of trust in government sustained the global infection. To deal with similar situations, like the pandemic, governments need support from other parties. On the one hand, to increase their credibility in front of citizens, policymakers need to have the public support of the healthcare authorities and research institutions by working together to find solutions and presenting them as a team to the affected population. On the other hand, policymakers need to reveal credible data to be trusted; thus, partnerships with researchers, data scientists, and data analysts during crises are indispensable. We can easily observe and state that during periods of general crisis (economic, medical, war), people are more willing to work together and share their knowledge and resources. Our results can be of interest to any of the parties mentioned above to develop our current state of research. Still, considering the topic, the general objective should be a collaboration as broad as possible to prevent a similar situation or to interpret the data collected so that we can generate solutions to similar problems.

The main limitation of the research consists of the unavailability of annual data on significant variables of COVID-19. Moreover, regarding the daily new confirmed deaths indicator, given the different protocols and changes in the causes of death, the number of deaths may not accurately present the actual number of deaths caused by COVID-19. Furthermore, in the case of the daily new confirmed cases indicator, given the limited testing, there is a possibility that the number of confirmed cases may be lower than the actual number in terms of COVID-19 infection. In regard to the share of daily positive COVID-19, issues related to testing reporting methods, but also differences in testing policies may affect comparison across countries. Hence, future research can focus on many other distinctive representative indicators from the health, digitalization, and COVID-19 dimensions. From these, we could mention the level of funding for healthcare (related to the digitalization process in the public healthcare system), citizens’ behavior (related to a healthy life, social life, or vaccination rate), and governance (related to trust in government or government efficiency) or e-Health indicators, being, of course, dependent on the data available in the future.

## Figures and Tables

**Table 1:** Description of the indicators.

Indicator	Definition	Unit of Measure	Source
Life expectancy at birth by sex	Life expectancy at birth—the mean number of years that a newborn child can expect to live.	Year	Eurostat
Live births and crude birth rate	Live births are the number of births of children who showed any sign that they are alive. Crude birth rate captures the ratio of the number of live births during the year to the average population related to that year.	Per 1000 persons	Eurostat
Median age	The median age divides the population into two equal parts, one category that includes people with ages below the median age, and the other category that includes people with ages above the median age.	Year	Our World in Data
Total fertility rate	Total fertility rate reflects the average number of live births per woman if the woman survives to the end of her reproductive life and experiences current age-specific fertility rates.	Live births per woman	Our World in Data
Health spending	Health spending comprises the current health expenditure, including personal health care and collective services.	Total, US dollars/capita	OECD
Connectivity—by mobile broadband	Reflects the demand and the supply side of fixed and mobile broadband.	Weighted score (0 to 100)	European Commission, Digital Scoreboard
Integration of digital technology—by digital intensity	It covers the measures of business digitisation and e-commerce, which also have a series of representative indicators, including digital intensity.	Weighted score (0 to 100)	European Commission, Digital Scoreboard
Digital public services—by e-government	Measures the demand and supply sides of digital public services and the open data.	Weighted score (0 to 100)	European Commission, Digital Scoreboard
Human capital—by internet user skills	It is measured by the number and complexity of activities that involve the use of the internet and digital devices.	Weighted score (0 to 100)	European Commission, Digital Scoreboard
Digital economy and society index, by aggregate score	DESI overall index, calculated as the weighted average of the four main DESI dimensions with an aggregate score.	Weighted score (0 to 100)	European Commission, Digital Scoreboard
Daily new confirmed COVID-19 cases per million people	The number of daily new conformed COVID-19 cases per million people.	7-day rolling average	Our World in Data (Data published by Johns Hopkins University CSSE COVID-19 Data)
Daily new confirmed COVID-19 deaths per million people	The number of daily new conformed COVID-19 deaths per million people.	7-day rolling average	Our World in Data (Data published by Johns Hopkins University CSSE COVID-19 Data)
Daily share of the population receiving a first COVID-19 vaccine dose	The percentage of the daily population receiving a first vaccine dose.	7-day rolling average (expressed as a percentage)	Our World in Data
The share of daily COVID-19 tests that are positive	The percentage of the daily positive COVID-19 tests.	7-day rolling average (expressed as a percentage)	Our World in Data

**Table 2:** Descriptive statistics, EU-27, 2020.

Variables	N	Mean	Standard Deviation	Min	Max
life_exp_birth	27	79.740	2.952	73.6	82.8
live_births_crude_birth	27	149,904.9	204,189.2	4414	773,144
median_age	27	43.077	2.337	37.3	47.9
fert_total	27	1.563	0.17	1.28	1.840
health_spend	27	3889.437	1577.356	1842.05	6730.94
human_cap_desi	27	27.313	6.421	13.35	39.24
connectivity_desi	27	15.422	1.991	12.63	19.8
int_digiteh_desi	27	6.661	2.846	1.27	12
digipubserv_desi	27	63.340	15.840	17.99	85.94
total_desi	27	47.626	9.122	29.98	62.8
daily_new_cases_cov	27	360.723	255.389	42.92	1009.6
daily_positive_tests_cov	27	12.458	8.787	1.08	35.25
daily_pop_fd_cov	27	0.185	0.498	0.01	2.3

Source: authors’ own process in Stata 17.

**Table 3:** Determination coefficients between DESI overall index (DESI Total) and DESI dimensions.

Independent	Human_cap_DESI	Connectivity_DESI	Int_DigiTeh_DESI	DigiPubServ_DESI
Coefficients	1.218 ***	1.929 **	2.754 ***	0.522 ***
R-square	0.7354	0.1773	0.7391	0.8233
t-Stat	8.336	2.321	8.417	10.793
F-test	69.496 ***	5.389 **	70.854 ***	116.50 ***

***, ** significant at 1%, respectively 5%.

**Table 4:** Division of countries into clusters in terms of health, K-means clustering algorithm method.

Clusters 0 (Green)	Cluster 1 (Pink)	Cluster 2 (Yellow)
Bulgaria	Austria	Belgium
Croatia	Germany	Cyprus
Czech Republic	Greece	Denmark
Estonia	Italy	Finland
Hungary	Netherlands	France
Latvia	Portugal	Ireland
Lithuania	Slovenia	Luxembourg
Poland	Spain	Malta
Romania		Sweden
Slovakia		

**Table 5:** Distribution of countries into clusters regarding digitalization indicators, K-means clustering algorithm method.

Clusters 0 (Red)	Cluster 1 (Orange)	Cluster 2 (Purple)
Greece	Austria	Bulgaria
Romania	Belgium	Croatia
	Denmark	Cyprus
	Estonia	Czech Republic
	Finland	Hungary
	France	Italy
	Germany	Poland
	Ireland	Slovakia
	Latvia	Slovenia
	Lithuania	
	Luxembourg	
	Malta	
	Netherlands	
	Portugal	
	Spain	
	Sweden	

**Table 6:** Distribution of countries into groups in terms of COVID-19 representative indicators, K-means clustering algorithm method.

Clusters 0 (Blue)	Cluster 1 (Brown)	Cluster 2 (Black)
Austria	Cyprus	Czech Republic
Belgium	Denmark	Lithuania
Bulgaria	Estonia	Slovakia
Croatia	Latvia	Slovenia
Finland	Netherlands	
France	Portugal	
Germany	Sweden	
Greece		
Hungary		
Hungary		
Ireland		
Italy		
Luxembourg		
Malta		
Poland		
Romania		
Spain		

## Data Availability

The raw data supporting the analysis and the conclusions of this paper can be made available by the authors, without undue reservation.
